# EBV-miR-BART5-5p regulates RORA to promote proliferation and migration of gastric cancer cells

**DOI:** 10.1371/journal.pone.0327323

**Published:** 2025-07-10

**Authors:** Changqi Du, Shuang Liang, Xia Wang, Yujiao Qi, Shangdong Li, Hongling Li

**Affiliations:** 1 Department of Oncology, Gansu Provincial Hospital, Lanzhou, Gansu, China; 2 The First Clinical Medical College, Gansu University of Chinese Medicine, Lanzhou, Gansu, China; 3 Clinical Medical College, Ningxia Medical University, Yinchuan, Ningxia, China; University of Nebraska-Lincoln, UNITED STATES OF AMERICA

## Abstract

**Background:**

Epstein-Barr virus-associated gastric cancer (EBVaGC) represents a distinct molecular subtype of gastric cancer. EBV encodes various viral RNAs, including BamHI-A rightward transcripts (BARTs), which are implicated in the carcinogenic processes of EBVaGC. This study aims to explore the function and underlying mechanisms of EBV-miR-BART5-5p in gastric cancer, providing a basis for the identification of more effective biomarkers for EBVaGC.

**Methods:**

Gene expression data were first downloaded from the GSE51575 dataset to identify differentially expressed genes and construct a WGCNA network, which led to the identification of RORA as a key gene associated with EBV-miR-BART5-5p. We then analyzed the TCGA dataset to investigate the differential expression and prognostic significance of RORA in gastric cancer. Further analysis explored RORA’s enriched pathways and its relationship with immune response, tumor mutation burden, and drug sensitivity. Single-cell gene expression characteristics of RORA were assessed using the GSE134520 dataset. RT-qPCR was employed to determine RORA expression levels in both EBV-positive and -negative gastric cancer cell lines. Western blotting and dual-luciferase reporter assays confirmed the targeting of RORA’s 3’ UTR by EBV-miR-BART5-5p. Finally, a series of functional experiments demonstrated that EBV-miR-BART5-5p promotes proliferation and migration of both EBV-positive and -negative gastric cancer cells.

**Results:**

In this study, differential expression and WGCNA analyses identified 910 co-expressed genes. We then investigated miR-BART5-5p in EBV-positive gastric cancer and identified RORA as a potential target gene. Our analysis revealed that RORA expression is lower in tumor samples compared to normal samples, and single-cell analysis showed significant upregulation of RORA in CD8 + T cells. Experimental data further demonstrated that RORA is expressed at lower levels in EBV-positive gastric cancer cell lines and that EBV-miR-BART5-5p targets the 3’ UTR of RORA. This suggests that EBV-miR-BART5-5p may promote gastric cancer cell proliferation and migration by regulating RORA.

**Conclusion:**

Our study reveals the molecular characteristics of EBV-associated gastric cancer, establishes a prognostic model for RORA in gastric cancer, and demonstrates that EBV-miR-BART5-5p may target and inhibit RORA to promote gastric cancer cell proliferation and migration. These findings highlight EBV-miR-BART5-5p could serve as a diagnostic biomarker and a potential therapeutic target for gastric cancer.

## 1. Introduction

Epstein-Barr Virus (EBV) was first identified in 1964 by Epstein and Barr in cultured cells derived from Burkitt lymphoma in African children. It is classified as Human Herpesvirus 4, specifically a γ-herpesvirus, known for its tropism for human lymphocytes [1]. In 1990, EBV was first detected in gastric carcinoma tissues using PCR, and it is now known that approximately 10% of gastric cancers worldwide are associated with EBV [2]. EBV-associated gastric cancer (EBVaGC) is a common malignancy linked to EBV infection [3]. Compared to EBV-negative gastric carcinoma (EBVnGC), EBVaGC is characterized by frequent PIK3CA mutations, widespread DNA hypermethylation, recurrent mutations in ARID1A and BCOR, and amplifications in genes such as JAK2 and PD-L1/2 [4]. In EBVaGC, the pattern of EBV latency is classified as Latency I or between Latency I and Latency II, with latent gene products including EBERs, EBNA1, and miR-BARTs, while LMP1 and LMP2B are either not expressed or expressed at low levels [5]. Additionally, EBV can induce DNA methylation in host cells, leading to abnormal gene expression and dysregulation of signaling pathways, thereby promoting the development of EBVaGC [6]. Clinically, EBVaGC is more common in males, tends to arise in the proximal or remnant stomach, and is associated with a lower incidence of lymph node metastasis and a more favorable prognosis [7]. These findings suggest that EBV-associated gastric cancer (EBVaGC) possesses distinct oncogenic characteristics and provide an opportunity to develop targeted therapies using EBV as a novel biomarker for gastric cancer.

MicroRNAs (miRNAs) are small non-coding RNAs that play crucial roles in post-transcriptional regulation [8]. EBV was the first virus identified to express miRNAs. Two distinct groups of EBV-miRNAs have been identified: BHRF1 and BART miRNAs [9]. miR-BARTs are expressed across all EBV latency types but are particularly abundant in epithelial cells, where they may target critical host epigenetic regulatory genes [10,11]. Research has shown that miR-BART11 can inhibit the expression of FOXP1, promoting epithelial-mesenchymal transition and thereby enhancing the invasive potential of tumor cells [12]. It has also been reported that miR-BART5-5p targets several genes associated with apoptosis and immune response, such as PUMA (p53 upregulated modulator of apoptosis) and MICB (MHC class I polypeptide-related sequence B) [13,14]. The role of miR-BART5-5p in EBV-associated gastric cancer (EBVaGC) remains to be further explored.

RORA (RAR-related orphan receptor A) is a transcription factor belonging to the orphan nuclear receptor family and has recently garnered significant attention in oncology research [15]. RORA regulates gene expression and is involved in various biological processes, including cell proliferation, differentiation, apoptosis, and immune responses [16]. Its expression and function are tightly regulated during tumorigenesis, and its role may vary depending on the type of tumor and microenvironment, exhibiting both oncogenic and tumor-suppressive properties [17,18]. Studies suggest that in estrogen receptor-positive (ER-positive) breast cancer, RORA may enhance cancer cell proliferation through the upregulation of aromatase expression, potentially contributing to tumorigenesis [18].In contrast, RORA displays tumor-suppressive activity in other cancer types. For instance, RORA expression is reduced in colorectal cancer tissues, and its low expression is associated with increased tumor invasiveness and poor prognosis [19–21]. RORA inhibits cancer cell proliferation and migration by suppressing the Wnt/β-catenin signaling pathway [17,20,22]. However, the mechanisms underlying RORA’s role in EBV-associated gastric cancer (EBVaGC) remain to be elucidated.

This study initially utilized bioinformatics analysis to identify differentially expressed genes (DEGs) between EBV-associated gastric cancer (EBVaGC) and EBV-negative gastric cancer (EBVnGC). Subsequently, Weighted Gene Co-expression Network Analysis (WGCNA) was employed to identify hub genes specific to EBVaGC, revealing co-expressed genes associated with DEGs. We explored the pathways potentially involved in the development of EBVaGC, and then investigated miR-BART5-5p in EBVaGC to identify its potential target gene, RORA. Analysis of the TCGA database was performed to evaluate RORA expression and its prognostic significance in gastric cancer, as well as its correlation with immune response, tumor mutational burden, and drug sensitivity. We also examined RORA gene characteristics at the single-cell level. Following this, a series of experimental validations were conducted in both EBV-positive and EBV-negative gastric cancer cell lines. Finally, we elucidated that miR-BART5-5p likely promotes gastric cancer cell proliferation and migration through the regulation of RORA. This study is significant for understanding the pathogenesis of EBVaGC and suggests that miR-BART5-5p and RORA may have potential as prognostic biomarkers.

## 2. Materials and methods

### 2.1 Data collection

The gene expression dataset GSE51575 was obtained from the NCBI GEO database (https://www.ncbi.nlm.nih.gov/geo/), comprising 12 EBV-positive gastric cancer tissues and 14 EBV-negative gastric cancer tissues. Gastric cancer data from The Cancer Genome Atlas (TCGA) can be accessed via (https://portal.gdc.cancer.gov/). This dataset includes next-generation sequencing data from 36 adjacent normal tissues and 412 gastric cancer tissues. After excluding patients with incomplete clinical information, 378 gastric cancer clinical samples were included in our study, with their clinical characteristics detailed in Table 1.To validate the expression levels of model genes across different cell subtypes, we retrieved single-cell RNA sequencing data (scRNA-seq) from the GEO database (http://www.ncbi.nlm.nih.gov/geo), specifically dataset GSE134520.

**Table 1:** Clinical features of GC patients in the TCGA dataset.

Clinical features	TCGA cohort
Status (%)	
Alive	230 (60.8%)
Dead	148 (39.2%)
Age (%)	
≤65	172 (45.5%)
>65	206 (54.5%)
Gender (%)	
Male	237 (62.7%)
Female	141 (37.3%)
Grade (%)	
G1	8 (2.1%)
G2	127 (33.6%)
G3	243 (64.3%)
WHO-Stage (%)	
Ⅰ	48 (12.7%)
Ⅱ	120 (31.7%)
Ⅲ	169 (44.7%)
Ⅳ	41 (10.8%)
AJCC-T stage (%)	
T1	17 (4.5%)
T2	76 (20.1%)
T3	180 (47.6%)
T4	105 (27.8%)
AJCC-N stage (%)	
N0	120 (31.7%)
N1	100 (26.5%)
N2	79 (20.9%)
N3	79 (20.9%)
AJCC-M stage (%)	
M0	351 (92.9%)
M1	27 (7.1%)

### 2.2 Identification of differentially expressed genes

First, we utilized R software (version 4.3.1) to read and process the GSE51575 dataset, applying batch correction and normalization. Differentially expressed genes (DEGs) were identified using the “LIMMA” package. Following the significance analysis of expression levels, we employed the “pheatmap” and “ggplot2” R packages to generate volcano plots and heatmaps of DEG expression.

### 2.3 Weighted gene co-expression network analysis

Weighted Gene Co-expression Network Analysis (WGCNA) is a systematic biological method frequently used to describe genetic association patterns among different samples. It is employed to identify highly correlated gene modules and to explore relationships between gene co-expression and phenotypes, thereby aiding in the identification of candidate biomarkers. We utilized the “WGCNA” R package to construct gene co-expression networks for EBV-positive and EBV-negative gastric cancer. Finally, we assessed the correlation between different modules and EBV infection, selecting the most relevant modules as core genes derived from the WGCNA analysis.

### 2.4 Selection of candidate key genes and GO/KEGG analysis

These intersecting genes were considered candidate hub genes associated with EBV infection. Subsequently, we performed Gene Ontology (GO) and KEGG pathway enrichment analyses using the “ClusterProfiler” R package. These analyses aimed to elucidate the potential mechanisms underlying disease progression and pathogenesis.

### 2.5 Protein-protein interaction analysis

We utilized the STRING database and the Cytoscape software platform to predict and visualize molecular interactions and protein-protein interaction (PPI) networks.

### 2.6 Bioinformatics prediction of target genes

We utilized three miRNA prediction databases—MicroT (http://diana.imis.athena-innovation.gr/DianaTools/index.php?r=MicroT_CDS/index), miRDB (https://mirdb.org/), and TarBase (https://dianalab.e-ce.uth.gr/tarbasev9)—to identify potential downstream target genes of EBV-miR-BART5-5p.

### 2.7 Identification of the RORA gene in gastric cancer

We used TIMER2.0 (http://timer.comp-genomics.org) to explore the pan-cancer expression levels of RORA between normal and tumor samples. Box plots were employed to display RORA expression levels in normal and tumor tissues. To validate differential expression of RORA between normal and tumor tissues, we obtained immunohistochemical staining images from the Human Protein Atlas (HPA) (https://www.proteinatlas.org/).

### 2.8 Correlation analysis of clinical features and construction of prognostic nomograms

To analyze the correlation between RORA expression levels and tumor clinical features such as patient age and staging, we performed χ² tests or Wilcoxon signed-rank tests. Significant results were validated using box plots. To develop a prognostic model for gastric cancer, we constructed a nomogram based on RORA expression levels, age, T stage, M stage, N stage, and clinical stage. Calibration curves for 1-year, 3-year, and 5-year survival rates were generated to evaluate the accuracy of the prognostic model.

### 2.9 Identification of RORA-related genes, pathways, and cellular functions in gastric cancer

To investigate the biological functions of RORA in gastric cancer, we conducted a correlation analysis between RORA and other genes using a threshold of |COR| > 0.5. The results were visualized with a Circos plot to illustrate genes highly correlated with RORA. Additionally, differential gene expression analysis was performed between high and low expression groups, with thresholds set at |LogFC| > 1 and FDR < 0.05. Gene Ontology (GO) and KEGG enrichment analyses were subsequently carried out to explore the molecular mechanisms and cellular functions associated with these differentially expressed genes.

### 2.10 Correlation of RORA with immune checkpoint-related genes and tumor mutational burden in gastric cancer

To evaluate the relationship between RORA and immune responses as well as tumor mutational burden in gastric cancer, we obtained expression data for immune checkpoint-related genes from the TCGA database. Correlation analyses were conducted to assess the relationship between RORA expression and immune checkpoint genes. Additionally, tumor mutational burden data from the TCGA gastric cancer samples were retrieved, and the correlation between RORA expression and tumor mutational burden was calculated.

### 2.11 Correlation analysis of RORA with drug sensitivity

To predict the potential benefits of chemotherapy for gastric cancer patients, we investigated the correlation between RORA expression levels and the IC50 values of commonly used chemotherapy drugs. IC50 data were downloaded from the GDSC database (https://www.cancerrxgene.org/), and the analysis was performed using the “pRRophetic” R package.

### 2.12 Analysis of single-cell sequencing data

We utilized the TISCH database (http://tisch.comp-genomics.org/) to examine the expression of RORA across various cell clusters in the single-cell dataset GSE134520 for gastric cancer.

### 2.13 Cell culture

The EBV-negative gastric cancer cell lines AGS and MKN-45, the EBV-positive gastric cancer cell line SNU719, and the human embryonic kidney cell line HEK293T were purchased from CELLCOOK (Guangzhou, China). HEK293T cells were cultured in Dulbecco’s Modified Eagle Medium (DMEM) supplemented with 10% fetal bovine serum (FBS) and 1% penicillin-streptomycin. Gastric cancer cells were cultured in RPMI-1640 medium containing 10% FBS and 1% penicillin-streptomycin. All cells were maintained in a humidified incubator at 37°C with 5% CO2.

### 2.14 Cell transfection

The EBV-miR-BART5-5p plasmid and the control plasmid PCDH were provided by Fudan University (China). To produce lentiviral particles, HEK293T cells were co-transfected with the miR-BART5-5p plasmid and packaging plasmids using Lipofectamine 3000 (Invitrogen, Carlsbad, California, USA). Lentivirus expressing miR-BART5-5p was used to transduce AGS and MKN-45 cells to establish stable cell lines. The EBV-miR-BART5-5p inhibitor and the negative control inhibitor (Inhibitor NC) were purchased from RiboBio (Guangzhou, China). Transfections into SNU719 cells were performed using Lipofectamine 3000 according to the manufacturer’s protocol.

### 2.15 Real-time quantitative PCR

Following the manufacturer’s instructions, total RNA was first extracted from cells using Trizol reagent (Invitrogen). cDNA was synthesized using the MonScript RTIII All-in-One Mix with dsDNase Kit (Monad Biotech Co., Ltd.). PCR analysis was subsequently performed using the SYBR Green qPCR Mix Kit (Monad Biotech Co., Ltd.). The primer sequences used were as follows: for miR-BART5-5p, the forward primer was 5’-CGTCAGCTGTCCGAGTAGAGGCAAGGTGAATATAGCTG-3’ and the reverse primer was 5’-TGTCAGGCAACCGTATTCACCCGATGGG-3’; for RORA, the forward primer was 5’-TAGCTCTTCAACACGTCCTACAG-3’ and the reverse primer was 5’-AGTCGCACAATGTCTGGGTATAT-3’; for U6, the forward primer was 5’-CTCGCTTCGGCAGCACA-3’ and the reverse primer was 5’-AACGCTTCACGAATTTGCGT-3’; and for GAPDH, the forward primer was 5’-GAAGGTGAAGGTCGGAGTC-3’ and the reverse primer was 5’-GAAGATGGTGATGGGATTTC-3’.

### 2.16 Protein western blot analysis

Total proteins were extracted from gastric cancer cells using RIPA buffer containing protease inhibitors. Protein concentration was determined using the BCA assay. Samples were separated by SDS-PAGE and transferred to PVDF membranes. The membranes were blocked with 5% milk for 1 hour and then incubated overnight at 4°C with the primary antibodies. Afterward, the membranes were incubated with HRP-conjugated secondary antibodies for 1 hour. Bands were detected using ECL and quantified with ImageJ. The primary antibodies used were RORA (Proteintech, China) and GAPDH (Zenbio, China), while the secondary antibody was anti-rabbit IgG (Immunoway, USA).

### 2.17 Cell proliferation, colony formation, and EdU assays

Cell proliferation was measured using the Cell Counting Kit-8 (CCK-8). Gastric cancer cells were seeded at a density of 1,500 cells per well in a 96-well plate and cultured in a 37°C, 5% CO₂ incubator for 24, 48, 72, and 96 hours. Subsequently, 10 μL of CCK-8 solution was added to each well and incubated for 2 hours. The absorbance was measured at 450 nm.For the colony formation assay, cells were seeded at a density of 1,000 cells per well in a 6-well plate and cultured for 14 days. Colonies were then fixed with 4% paraformaldehyde and stained with 1% crystal violet for 30 minutes. The average number of colonies was quantified using ImageJ software.For the EdU assay, the procedure was followed according to the instructions provided in the EdU detection kit (Beyotime).

### 2.18 Wound healing assay and transwell migration assay

For the wound healing assay, cells were seeded at a density of 4 × 10^5 cells per well in a 6-well plate and cultured until they reached approximately 90–100% confluence. Once confluent, a straight-line scratch (wound) was created in the cell monolayer using a sterile 200 μL pipette tip. The wells were gently washed twice with phosphate-buffered saline (PBS) to remove detached cells. Images were captured at 0 hours and 24 hours post-wounding under a microscope to assess wound healing.For the Transwell migration assay, a 24-well Transwell chamber with an 8 μm pore size polycarbonate membrane (Corning, New York, USA) was used. Gastric cancer cells (1 × 10^4) were suspended in serum-free RPMI 1640 medium and seeded in the upper chamber, while the lower chamber contained 700 µL of RPMI 1640 medium with 10% FBS. After incubation at 37°C for 24 hours, cells adhering to the underside of the Transwell membrane were fixed with 4% paraformaldehyde at room temperature for 20 minutes, stained with 0.1% crystal violet for 15 minutes, and visualized under a microscope. Migration cells were quantified using ImageJ software.

### 2.19 Luciferase reporter assay

Lipofectamine 2000 (Invitrogen, USA) was used to co-transfect gastric cancer cells with the firefly luciferase reporter plasmid and the Renilla luciferase control plasmid. After 24–48 hours, cells were lysed, and luciferase activity was measured using the Dual-Luciferase Reporter Assay System according to the manufacturer’s instructions. Firefly luciferase activity was normalized to Renilla luciferase activity. All experiments were performed in triplicate.

### 2.20 Statistical analysis

Data processing, analysis, and visualization were performed using R software and relevant packages (version 4.3.1). Experimental data analysis was conducted using GraphPad Prism. Statistical significance was defined as a p-value < 0.05 for all tests to determine the significance of the results.

## 3. Results

### 3.1 Differential gene screening

**Fig1:**
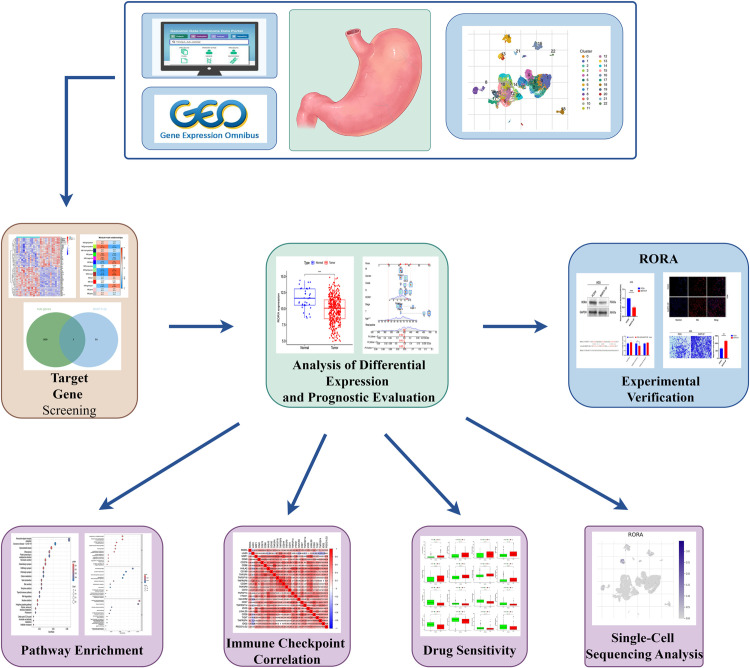
Flowchart of the study © 2024 by Figdraw is licensed under CC BY 4.0. To view a copy of this license, visit https://creativecommons.org/licenses/by/4.0/.

**Fig 2:**
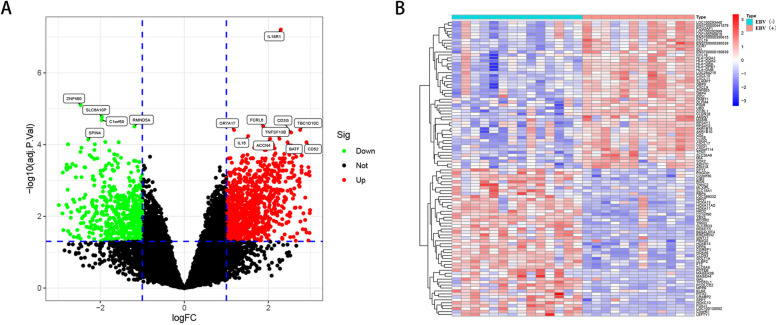
Identification of DEGs between EBV-positive and EBV-negative gastric cancer. (A) Volcano plot of differential expression analysis from the GSE51575 dataset.(B) Heatmap of differential expression analysis from the GSE51575 dataset. Green represents downregulated genes, red represents upregulated genes, and black represents non-differentiated genes.

The workflow of this study is illustrated in Fig 1. We first accessed the GSE51575 dataset comprising Epstein–Barr virus (EBV)-positive and EBV-negative gastric cancer samples. Differential expression analysis identified 1,583 differentially expressed genes (DEGs) between EBV-positive and EBV-negative groups, including 994 upregulated and 589 downregulated genes (|log FC| ≥ 1, p < 0.05) (Fig 2A and 2B).

### 3.2 WGCNA network construction and identification of EBV infection-related modules

**Fig3:**
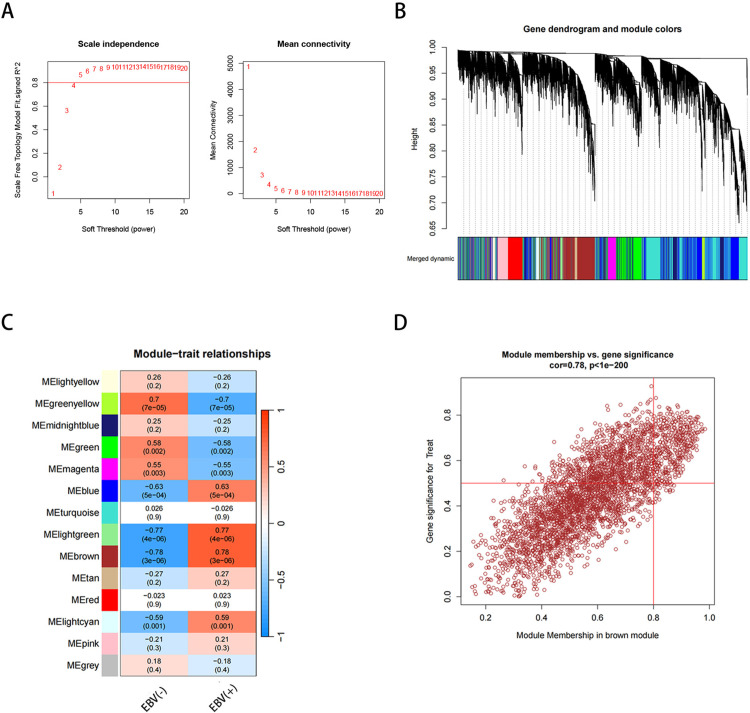
Identification of EBV infection-related gene modules using WGCNA. (A) Analysis of the scale-free topology index and mean connectivity for various soft-thresholding powers. (B) Dendrogram of differentially expressed genes based on dissimilarity measures (1-TOM) clustering. (C) Heatmap showing the correlation between modules and trait-related gene sets. Each module is labeled with the corresponding correlation coefficient and p-value. (D) Scatter plot analysis of the brown module.

To investigate gene networks associated with Epstein–Barr virus (EBV) infection, we performed a WGCNA analysis. A soft-thresholding power of 5 was selected to achieve a scale-free topology with optimal network connectivity(Fig 3A). Hierarchical clustering of the topological overlap matrix identified 14 distinct gene modules, each denoted by a unique colour (Fig 3B). Among these gene modules, the brown module showed a strong correlation with EBV infection (correlation coefficient of 0.78, p-value of 3 × 10^-6, Fig 3C). Thus, the brown module was selected as the hub module, containing 3333 genes (Fig 3D).

### 3.3 Screening of co-expressed genes, GO/KEGG analysis, and PPI network analysis

**Fig 4:**
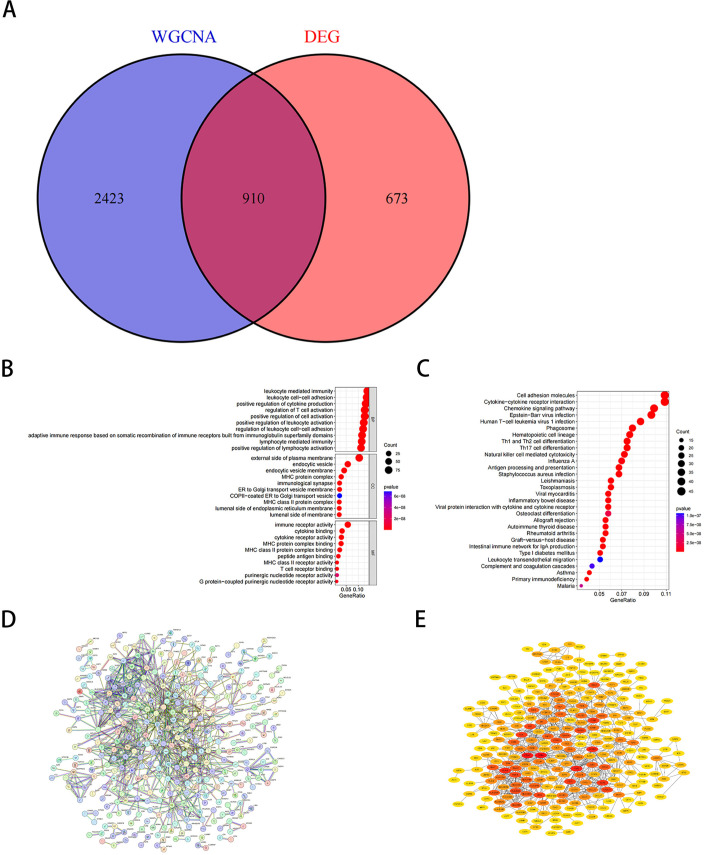
Screening of candidate hub genes, functional enrichment analysis, and construction of the PPI network. (A) Venn diagram showing 910 overlapping candidate hub genes. (B) GO enrichment analysis of candidate hub genes. (C) KEGG pathway analysis of candidate hub genes. (D) PPI network of overlapping hub genes. (E) Core genes identified within the interaction network using the degree algorithm.

To identify genes potentially implicated in the pathogenesis of EBV-positive gastric cancer, we intersected the differentially expressed genes (DEGs) with those derived from the WGCNA hub module, yielding 910 overlapping candidates (Fig 4A). Gene Ontology (GO) and Kyoto Encyclopedia of Genes and Genomes (KEGG) pathway analyses were subsequently performed to investigate the functional relevance of these candidate hub genes.GO enrichment analysis revealed that the candidate hub genes were significantly enriched in biological processes related to positive regulation of leukocyte-mediated immunity, leukocyte adhesion, and cytokine production. In terms of cellular components, the candidate hub genes were predominantly enriched in the extracellular side of the plasma membrane, intracellular vesicles, and endosomal vesicle membranes. For molecular functions, these genes were rich in immune receptor activity, cytokine binding, and cytokine receptor activity (Fig 4B). Additionally, KEGG analysis indicated that the candidate hub genes were significantly enriched in pathways related to cell adhesion molecules, cytokine-cytokine receptor interactions, and chemokine signaling (Fig 4C).Subsequently, we used the STRING online tool to construct a protein-protein interaction (PPI) network for the overlapping hub genes (Fig 4D), and visualized this network using Cytoscape software (Fig 4E).

### 3.4 Identification of downstream target genes of EBV-miR-BART5-5P

First, we predicted the downstream target genes of EBV-miR-BART5-5p using three databases: MicroT, MiRDB, and TarBase, and identified 17 candidate genes through intersection analysis. We then intersected these with the candidate hub genes to identify the characteristic genes, revealing RORA as a potential target of EBV-miR-BART5-5p (S1 Fig).

### 3.5 Identification of differential expression of RORA in gastric cancer

**Fig 5:**
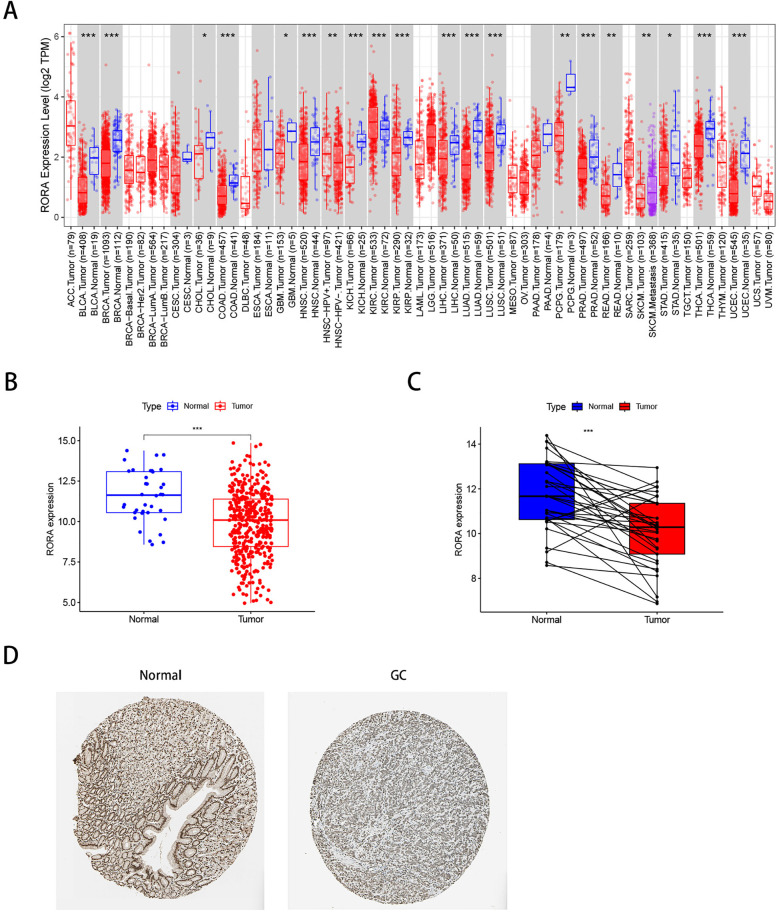
Differential expression of the characteristic gene RORA. (A) Pan-cancer analysis of RORA expression in the TIMER database, with red and blue representing tumor and normal samples, respectively. (B) mRNA expression of RORA in STAD from the TCGA database. (C) mRNA expression of RORA in paired tumor and adjacent normal tissues of gastric cancer patients from the TCGA database. (D) Protein expression of RORA in gastric cancer and normal gastric tissues from the HPA database.

We first analyzed the expression of RORA across various cancer types (Fig 5A). RORA levels were found to be lower in tumor samples compared to normal samples in several cancers, including bladder urothelial carcinoma, invasive breast carcinoma, cholangiocarcinoma, colon cancer, glioblastoma multiforme, head and neck squamous cell carcinoma, clear cell renal carcinoma, papillary renal cell carcinoma, hepatocellular carcinoma, lung adenocarcinoma, squamous cell lung carcinoma, pheochromocytoma, paraganglioma, prostate cancer, rectal cancer, gastric cancer, thyroid carcinoma, and endometrial carcinoma. In contrast, RORA expression was lower in normal samples compared to renal clear cell carcinoma tumor samples. In gastric cancer, RORA expression was significantly reduced in tumor tissues compared to normal tissues (Fig 5B). Similar decreases in RORA expression were observed in GC tumor tissues compared to adjacent non-tumor tissues (Fig 5C). Additionally, immunohistochemical analysis from HPA showed that RORA expression was lower in tumor tissues compared to non-tumor tissues (Fig 5D).

### 3.6 Clinical pathological features analysis and construction and validation of nomograms

**Fig 6:**
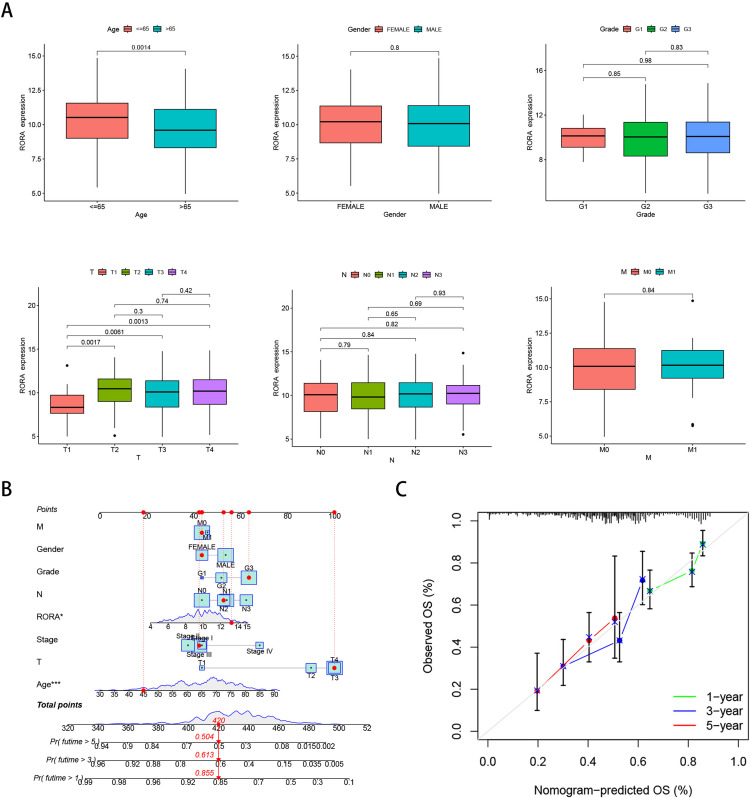
Analysis of clinical features and construction of the nomogram model. (A) Relationship between age and RORA expression. (B) Relationship between cancer stage and RORA expression. (C) Construction of the RORA nomogram model in TCGA. (D) Calibration curve of the nomogram.

To analyze the correlation between RORA expression and clinical features, we compared the expression of RORA across different clinical subgroups. The results revealed significant differences in RORA expression based on age and clinical staging (Fig 6A). Subsequently, given the strong association between risk scores and patient outcomes, we incorporated clinical parameters to construct a nomogram for estimating the 1-year, 3-year, and 5-year overall survival (OS) in gastric cancer (GC) patients (Fig 6B). The calibration curves of the nomogram demonstrated high accuracy in predicting OS, aligning closely with actual observed values (Fig 6C).

### 3.7 Identification of RORA-related genes in gastric cancer and GO/KEGG analysis

**Fig 7:**
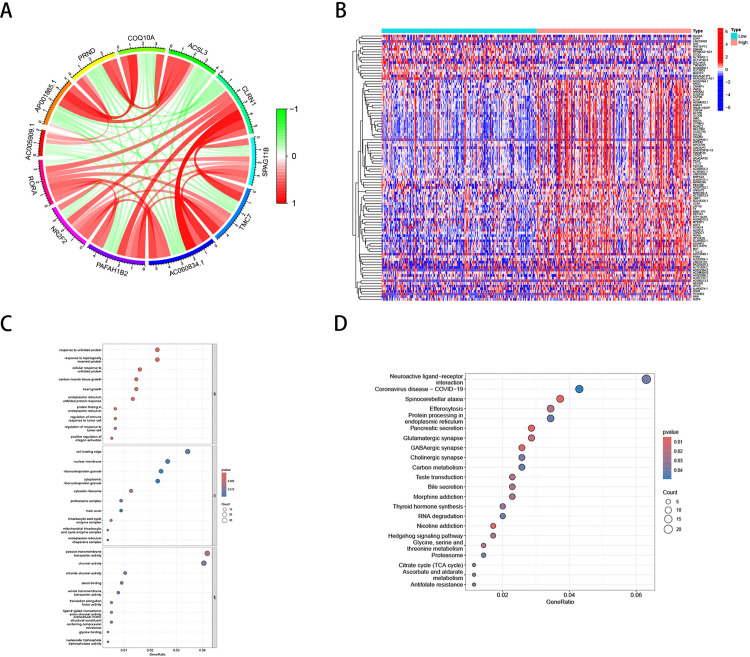
Identification of RORA-related genes and functional enrichment analysis. (A) Analysis of RORA-related genes in TCGA. (B) Heatmap showing differential gene expression between high and low RORA expression groups. (C) GO analysis of differential genes associated with RORA. (D) KEGG pathway analysis of differential genes associated with RORA.

Based on RNA sequencing data from the TCGA database, co-expression analysis identified 47 genes significantly associated with RORA. Fig 7A illustrates the interactions between RORA and 11 genes highly correlated with RORA. Between the high and low RORA expression groups, we identified 1,326 differentially expressed genes (DEGs), with 1,310 genes upregulated and 16 genes downregulated in the high expression group (Fig 7B).GO enrichment analysis revealed that DEGs were significantly enriched in biological processes related to the response to unfolded proteins, response to topologically incorrect proteins, and cellular response to unfolded proteins. In terms of cellular components, DEGs were mainly enriched in cell periphery, nuclear envelope, and nuclear proteins. For molecular functions, DEGs were notably enriched in passive transmembrane transporter activity and channel activity (Fig 7C). Additionally, KEGG analysis showed significant enrichment of DEGs in pathways related to neuroactive ligand-receptor interactions, COVID-19, and spinocerebellar ataxia (Fig 7D).

### 3.8 Correlation analysis of RORA with immunity and tumor mutation burden in gastric cancer

We analyzed the correlation between RORA expression and immune checkpoint-related genes, as well as tumor mutation burden in gastric cancer. The results showed that in gastric cancer, RORA expression was positively correlated with the expression of NRP1, CD40, CD276, CD86, HHLA2, CD160, TNFSF4, TNFSF18, TNFRSF8, CD244, TNFSF9, CD70, TNFSF14, VTCN1, HAVCR2, CD27, TNFRSF14, ICOS, LGALS9, CD28, TIGIT, TNFRSF4, IDO2, and PDCD1LG2, while it was negatively correlated with LAG3 expression. Tumor mutation burden analysis revealed a significant negative correlation between RORA expression and tumor mutation burden in gastric cancer(S2 Fig).

### 3.9 Drug sensitivity analysis

**Fig 8:**
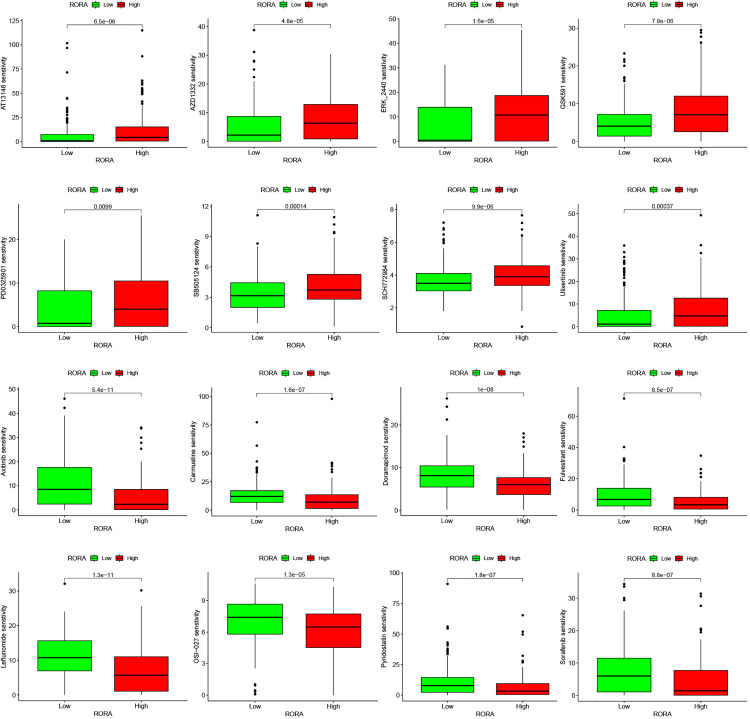
Drug sensitivity analysis between high and low expression groups.

Next, we performed differential analysis of the half-maximal inhibitory concentration (IC50) values of commonly used chemotherapeutic agents between the RORA high-expression and low-expression groups. The results indicated that the low-expression group had lower IC50 values for several anticancer drugs, including AT13148, AZD1332, ERK_2440, GSK591, PD0325901, SB505124, SCH772984, and Urolithin. In contrast, the high-expression group exhibited lower IC50 values for other anticancer drugs such as Axitinib, Carmustine, DMM, Fulvestrant, Lenvatinib, OSI-027, Trichostatin A, and Sorafenib (Fig 8).

### 3.10 Validation of gene features in gastric cancer scRNA-seq data

**Fig 9:**
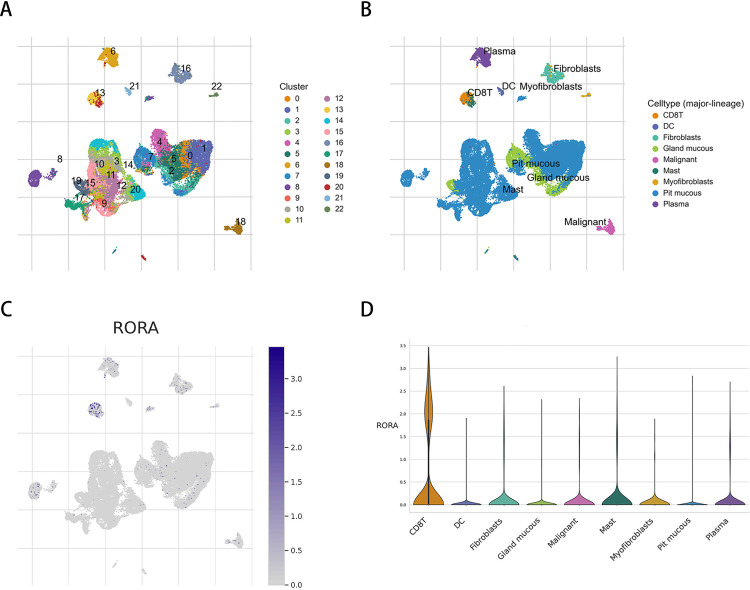
Gene expression in single-cell gastric cancer datasets. (A) Identification of 23 clusters using UMAP.(B) Visualization of 9 distinct cell subtypes.(C) UMAP plot showing the expression of specific genes.(D) Violin plots depicting gene expression across different cell types.

Due to significant differences between cell subtypes, we selected the gastric cancer single-cell dataset (GSE134520) from the TISCH database to investigate the distribution of RORA across different cell subtypes. After performing dimensionality reduction and clustering using UMAP analysis, we identified a total of 22 distinct cell clusters (Fig 9A). Based on the subtype annotations, we determined 9 major cell types, including CD8 + T cells, dendritic cells, fibroblasts, glandular cells, malignant cells, mast cells, myofibroblasts, small crater cells, and plasma cells (Fig 9B). We then analyzed RORA expression across these cell subtypes to differentiate them (Fig 9C). The results indicated that RORA expression was significantly upregulated in CD8 + T cells within gastric cancer (Fig 9D).

### 3.11 Assessment of RORA expression in gastric cancer cell lines

We selected the EBV-negative gastric cancer cell lines AGS and MKN-45, alongside the EBV-positive gastric cancer cell line SNU719, for experimental studies. RNA was extracted, and RORA expression levels were measured using RT-qPCR. The results revealed significantly higher RORA expression in AGS and MKN-45 compared to SNU719, indicating a notable difference in RORA expression between EBV-negative and EBV-positive gastric cancer cell lines (S3 Fig).

### 3.12 EBV-miR-BART5-5p targets RORA and inhibits its expression in gastric cancer cells

**Fig 10:**
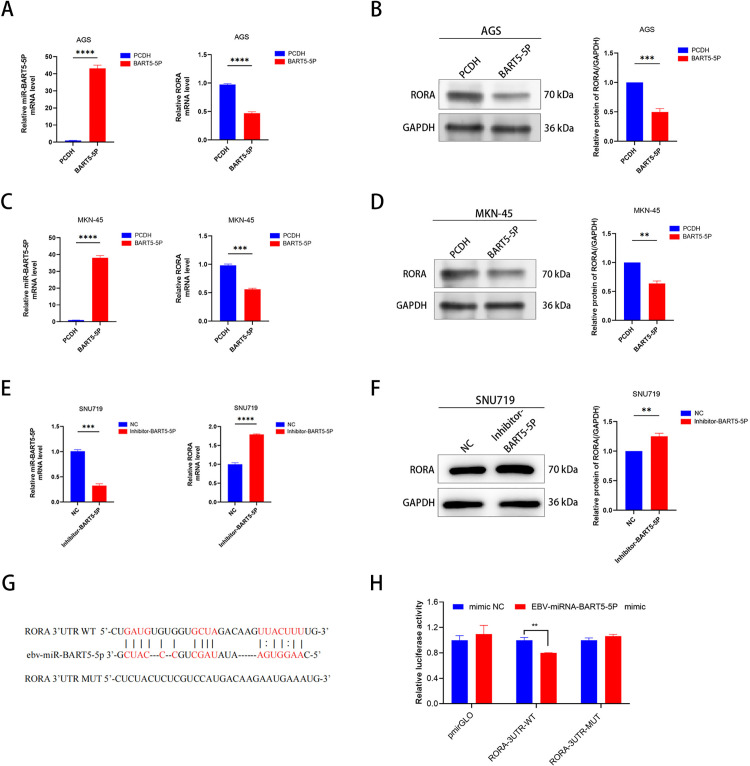
EBV-miR-BART5-5p targets and inhibits RORA expression. (A-F) Detection of EBV-miR-BART5-5p and RORA expression in negative GC cell lines (AGS, MKN-45) and positive GC cell line (SNU719) using PCR and Western blotting.(G) Bioinformatics prediction of binding sites for BART5-5p in the RORA 3’ UTR, showing wild-type (WT) and mutant (MUT) sequences.(H) Dual-luciferase reporter assays. HEK293T cells were co-transfected with reporter vectors containing either WT or MUT RORA 3′-UTR, along with EBV-miR-BART5-5p mimics or negative control (nc).

Bioinformatics analysis suggests that RORA may be regulated by EBV-miR-BART5-5p.To investigate how miR-BART5-5p modulates RORA expression, we conducted overexpression experiments in AGS and MKN-45 cell lines. We assessed RORA expression using real-time quantitative PCR (RT-qPCR) and Western blotting. The results indicated that, compared to the PCDH blank control group, RORA mRNA and protein levels were significantly reduced in the miR-BART5-5p overexpression group for both AGS and MKN-45 cell lines (Fig 10A–10D), with statistical significance.Subsequently, we performed miR-BART5-5p knockdown experiments in the SNU719 cell line. Compared to the NC control group, RORA mRNA and protein levels were significantly increased in the miR-BART5-5p knockdown SNU719 cells (Fig 10E–10F), with statistical significance.To determine whether miR-BART5-5p directly targets the RORA 3’ UTR, we introduced mutations at the predicted binding sites within the RORA 3’ UTR and generated a mutant RORA 3’ UTR reporter construct (Fig 10G). As anticipated, treatment with the miR-BART5-5p mimic led to a reduction in luciferase activity of the wild-type RORA 3’ UTR reporter, whereas the mutant reporter exhibited no such reduction (Fig 10H). Collectively, these findings suggest that EBV-encoded miR-BART5-5p downregulates RORA expression by directly targeting its 3’ UTR.

### 3.13 EBV-miR-BART5-5p promotes proliferation and migration of gastric cancer cells

**Fig 11:**
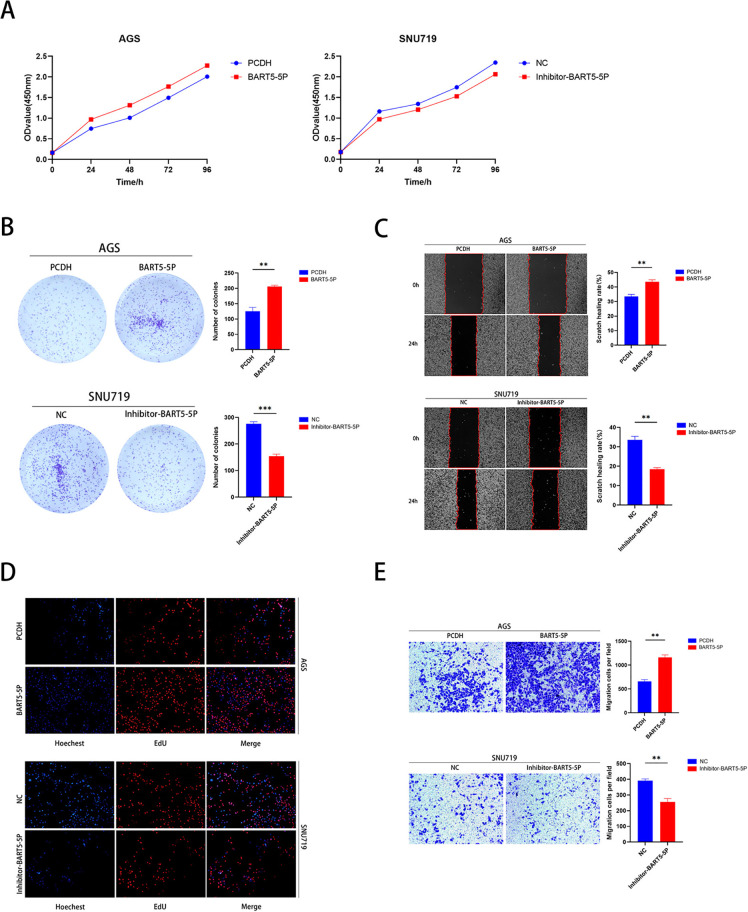
EBV-miR-BART5-5p promotes proliferation and migration in gastric cancer cells. (A) Cell viability in AGS and SNU719 cell lines was assessed using the CCK-8 assay.(B) Clonogenic assays were performed to evaluate the proliferation capacity of AGS and SNU719 cells. (C) Wound healing assays were conducted to assess the migration ability of AGS and SNU719 cells. (D) The EdU incorporation assay was used to measure cell proliferation in AGS and SNU719 cells. (E) Transwell migration assays were employed to evaluate the migration capacity of AGS and SNU719 cells.

To further explore the role of EBV-miR-BART5-5p in gastric cancer cells, we conducted experiments using both EBV-negative AGS and EBV-positive SNU719 cell lines. Initially, we stably transfected AGS cells with PCDH and miR-BART5-5p. The effects on cell proliferation and migration were assessed using CCK-8 assays, colony formation assays, wound healing assays, EdU assays, and Transwell assays. The results indicated that upregulation of EBV-miR-BART5-5p significantly enhanced both proliferation and migration of gastric cancer cells compared to the PCDH control group. Subsequently, we transfected SNU719 cells with miR-BART5-5p inhibitors and negative controls. Similar assays were performed, revealing that downregulation of EBV-miR-BART5-5p inhibited cell proliferation and migration compared to the negative control group (Fig 11A–11E). These findings suggest that EBV-miR-BART5-5p enhances proliferation and migration of gastric cancer cells.

## 4. Discussion

In EBV-associated tumors, epithelial tumors account for over 80%, with the majority being nasopharyngeal carcinoma and EBV-associated gastric carcinoma (EBVaGC) [23]. EBVaGC is a distinct subtype of gastric cancer with different molecular characteristics, biological behaviors, and clinical presentations compared to other types of gastric cancer [24,25].In this study, differential expression analysis and Weighted Gene Co-Expression Network Analysis (WGCNA) were performed on the GSE51575 dataset to identify candidate hub genes for EBVaGC. Subsequently, Gene Ontology (GO) and Kyoto Encyclopedia of Genes and Genomes (KEGG) pathway enrichment analyses, along with protein-protein interaction (PPI) network construction, revealed that the candidate hub genes for EBVaGC were significantly enriched in pathways related to cell adhesion molecules, cytokine-cytokine receptor interactions, and chemokine signaling. These bioinformatics analyses uncovered complex interactions between various biological processes and pathways, providing valuable insights into the biological mechanisms underlying EBVaGC.

EBV-encoded microRNAs (miRNAs), particularly those from the BamHI A rightward transcripts (BART) family, play a crucial role in EBV-associated cancers [5,23]. BART miRNAs can target both viral genes and several key regulatory factors within host cells [26,27]. Due to their high expression in EBV-related cancers, BART miRNAs have potential as biomarkers for disease diagnosis and prognosis [28,29]. Targeting BART miRNAs or their target genes with small molecules, antibody drugs, or RNA interference strategies is currently being explored as a therapeutic option [30].There is increasing evidence that BART miRNAs are involved in various biological processes in EBV-associated gastric cancer (EBVaGC), including evasion of the host immune system, latency establishment, cell proliferation, apoptosis, and metastasis [31,32]. Elevated levels of BART miRNAs and the loss of the BHRF1 cluster have been reported in EBVaGC cell lines and patient samples [33,34]. The high expression of BART miRNAs suggests their significant role in the transformation of gastric epithelial cells [35].Shinozaki et al. analyzed the expression of 44 known EBV miRNAs in human tissues and EBVaGC cell lines, identifying potential target genes implicated in tumorigenesis, tumor suppression, adhesion pathways, and apoptosis, including genes from the Bcl-2 family [33]. They validated the anti-apoptotic effect of miR-BART4-5p by downregulating Bid expression in EBV-positive cell lines [33]. Zheng et al. demonstrated that miR-BART5-3p directly targets and inhibits the tumor suppressor gene TP53, downregulating the cell cycle inhibitor p21 and thereby promoting tumor development [36]. Similarly, miR-BART3-3p regulates SASP expression to inhibit NK cell and macrophage infiltration in tumor tissue, aiding gastric cancer cell immune evasion [37]. Moreover, miR-BART10-3p and miR-BART22 activate the Wnt signaling pathway, thereby enhancing tumor cell migration [38]. Further studies are required to fully elucidate the precise roles of BART miRNAs in the pathogenesis of EBVaGC.

In recent years, miR-BART5-5p has garnered significant attention in the study of EBV-associated cancers. miR-BART5-5p exerts its regulatory effects on cellular functions by binding to mRNA targets within host cells, thereby suppressing their expression [39]. PUMA, a pro-apoptotic protein, is directly targeted and inhibited by miR-BART5-5p, enabling EBV to reduce the apoptotic sensitivity of host cells, which promotes cell survival and viral latency post-infection [36]. MICB, a critical marker for natural killer (NK) cell recognition and clearance of virus-infected cells, is also suppressed by miR-BART5-5p, aiding EBV-infected cells in evading NK cell-mediated immune surveillance [40].However, the role of miR-BART5-5p in EBVaGC requires further investigation. Shinozaki et al. [33]found elevated miR-BART5-5p expression levels in tissue samples from EBVaGC patients. Kim et al. [41]analyzed miR-BART5 expression in EBVaGC tissues and cell lines, revealing its indispensable role in the progression of EBV-related gastric cancer. In this study, we explored the biological function of miR-BART5-5p in EBVaGC and discovered that miR-BART5-5p might promote gastric cancer cell proliferation and migration by regulating RORA. Therefore, miR-BART5-5p holds potential as a biomarker for predicting gastric cancer progression and treatment response.

RORA (RAR related orphan receptor A) is a transcription factor belonging to the nuclear receptor superfamily, playing a pivotal role in various biological processes, including immune response, metabolic regulation, neurodevelopment, and cell proliferation [42,43]. As a transcriptional activator, RORA regulates the expression of downstream genes by directly binding to specific DNA sequences [44]. In recent years, the role of RORA in multiple diseases, particularly in inflammatory diseases, metabolic disorders, neurological conditions, and cancers, has garnered considerable attention [15]. Research has shown that RORA potentially influences cancer progression by regulating the cell cycle, modulating apoptosis, and impacting immune responses within the tumor microenvironment [45].The role of RORA varies across different types of cancers; it can function either as a tumor promoter or as a tumor suppressor. For instance, elevated RORA expression has been associated with increased invasiveness and poor prognosis in specific breast cancer subtypes [18]. Conversely, in other cancer types, low RORA expression may be linked to tumor growth and metastasis [46]. In hepatocellular carcinoma, diminished RORA expression is associated with tumor growth, invasion, and poor prognosis [47,48]. Furthermore, RORA can modulate immune responses, enhancing the immune system’s ability to eliminate tumor cells [49,50]. These differences in RORA’s role may be attributed to varying expression levels, distinct downstream target genes, and the influence of the tumor microenvironment across different cancer types [51].

This study, through pan-cancer analysis and TCGA-STAD dataset analysis, revealed that RORA expression levels in gastric cancer (GC) tissues are significantly lower than in normal tissues, a finding further validated by immunohistochemical staining in the Human Protein Atlas (HPA) database. Clinical pathological feature analysis and nomogram construction were utilized to predict 1-year, 3-year, and 5-year overall survival (OS) for GC patients, highlighting the association between RORA expression and patient prognosis. Further analysis demonstrated that RORA expression in gastric cancer is positively correlated with immune checkpoint-related genes, including NRP1 and CD40. Previous studies suggest that activating or enhancing RORA expression may strengthen antitumor immune responses and improve the effectiveness of existing immunotherapies, such as immune checkpoint inhibitors [52].Our analysis also revealed a significant inverse correlation between RORA expression and tumor mutational burden (TMB). The importance of TMB is broadly recognized in solid tumors, where it can serve as part of a composite biomarker for predicting immune checkpoint inhibitor outcomes [53]. Drug sensitivity analysis indicated that patients with high RORA expression had lower IC50 values for anticancer drugs such as axitinib and carmustine. Axitinib reduces tumor vascularization, depriving tumors of essential nutrients and oxygen necessary for proliferation [54]. Carmustine (BCNU) exerts its antitumor effects by crosslinking DNA strands, leading to disrupted DNA replication and transcription, ultimately causing cell cycle arrest and apoptosis in rapidly dividing tumor cells [55]. The observed low IC50 values suggest these drugs may be potent tools against cancer.At the single-cell level, we found that RORA expression is significantly upregulated in CD8 + T cells within GC tissues. The survival and functional efficacy of CD8 + T cells can enhance cancer treatment outcomes [56]. In this study, differences in RORA expression were observed between EBVaGC and EBV-negative GC cells. Further investigation is needed to elucidate the specific mechanisms of RORA in gastric cancer and to assess its potential as a therapeutic target in clinical settings.

Additionally, we confirmed that miR-BART5-5p directly targets and inhibits the 3’UTR of RORA through Western blot and dual-luciferase reporter assays. To assess the impact of miR-BART5-5p on cell proliferation and migration, we conducted CCK-8 assays, colony formation assays, EdU assays, wound healing assays, and Transwell assays. The results revealed that miR-BART5-5p enhances the proliferation and migration of gastric cancer (GC) cells. In summary, our database analysis and experimental results provide strong evidence that EBV-miR-BART5-5p may promote gastric cancer cell proliferation and migration by targeting and inhibiting RORA. These findings suggest that EBV-miR-BART5-5p could serve as a therapeutic biomarker, with RORA being a promising target for the treatment of EBV-associated gastric carcinoma (EBVaGC).

## Supporting information

S1 Fig Venn diagram of target gene bioinformatics predictions. (TIF)

S2 Fig Analysis of the correlation between RORA, immunity, and tumor mutation burden. (TIF)

S3 Fig RORA expression in SNU719, AGS, and MKN-45 cell lines. (TIF)

S1 File Original western blot image. (DOCX)

## Abbreviations

BARTs: BamHI A rightward transcripts
DEGs: Differentially expressed genes
DMEM: Dulbecco’s Modified Eagle Medium
EBVaGC: EBV-Associated Gastric Cancer
GC: Gastric Cancer
GEO: Gene Expression Omnibus Database
GO: Gene ontology
HPA: the Human Protein Atlas
KEGG: Kyoto Encyclopedia of Genes and Genomes
miRNAs: MicroRNAs
PPI: Protein-protein interaction
RORA: RAR-related orphan receptor A
RT-qPCR: Quantitative Real-time Reverse Transcription Polymerase Chain Reaction
TCGA: The Cancer Genome Atlas
TMB: Tumor mutational burden
WB: Western Blot
WGCNA: Weighted Gene Co-expression Network Analysis
