# Supplementary figures and images for: EBV-miR-BART5-5p regulates RORA to promote proliferation and migration of gastric cancer cells
Source: PLoS One. 2025 Jul 10;20(7):e0327323. doi: 10.1371/journal.pone.0327323 (PMC12244631; doi:10.1371/journal.pone.0327323)

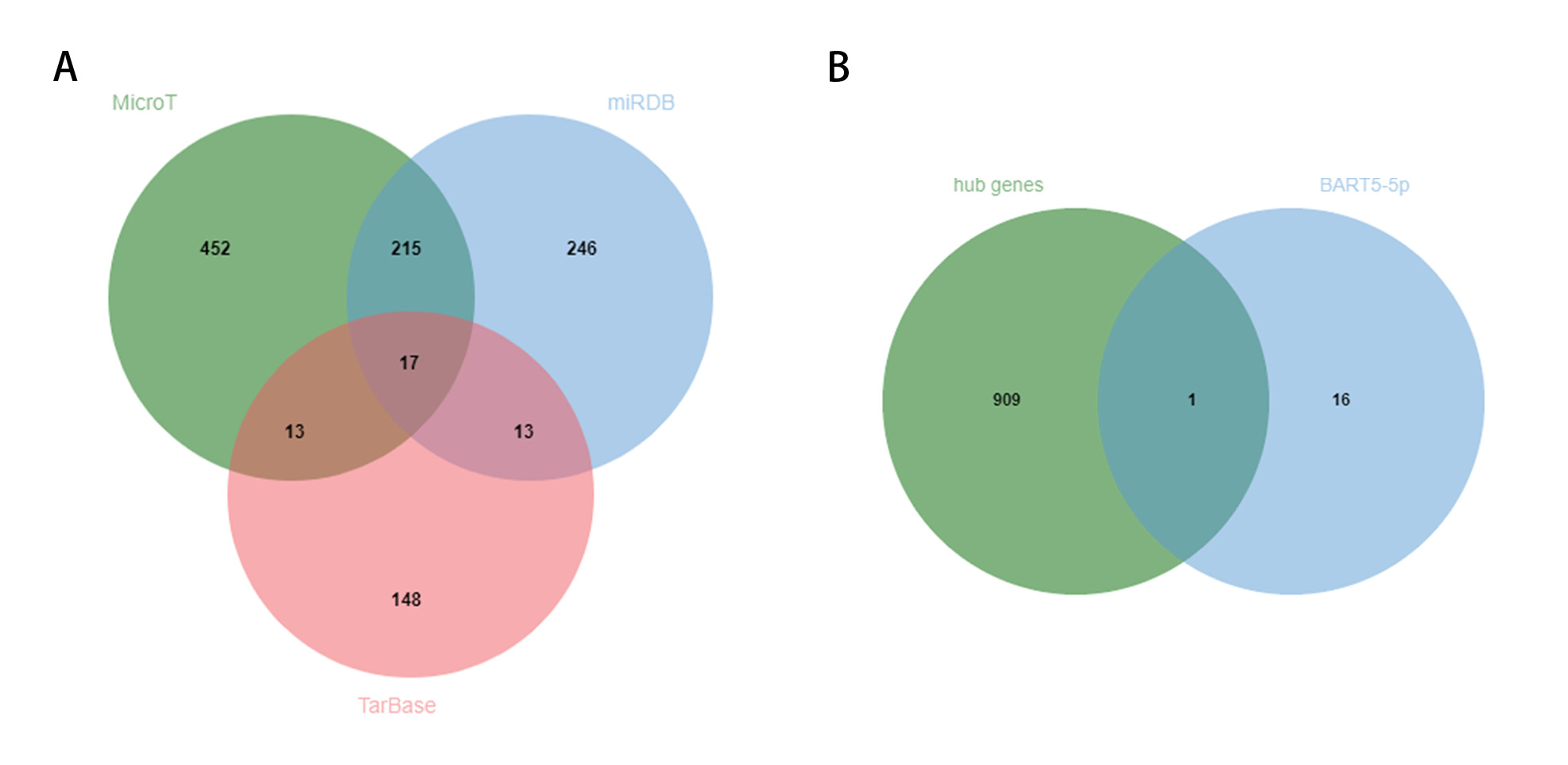

Supplement: S1 Fig — (TIF) [file pone.0327323.s001.tif]

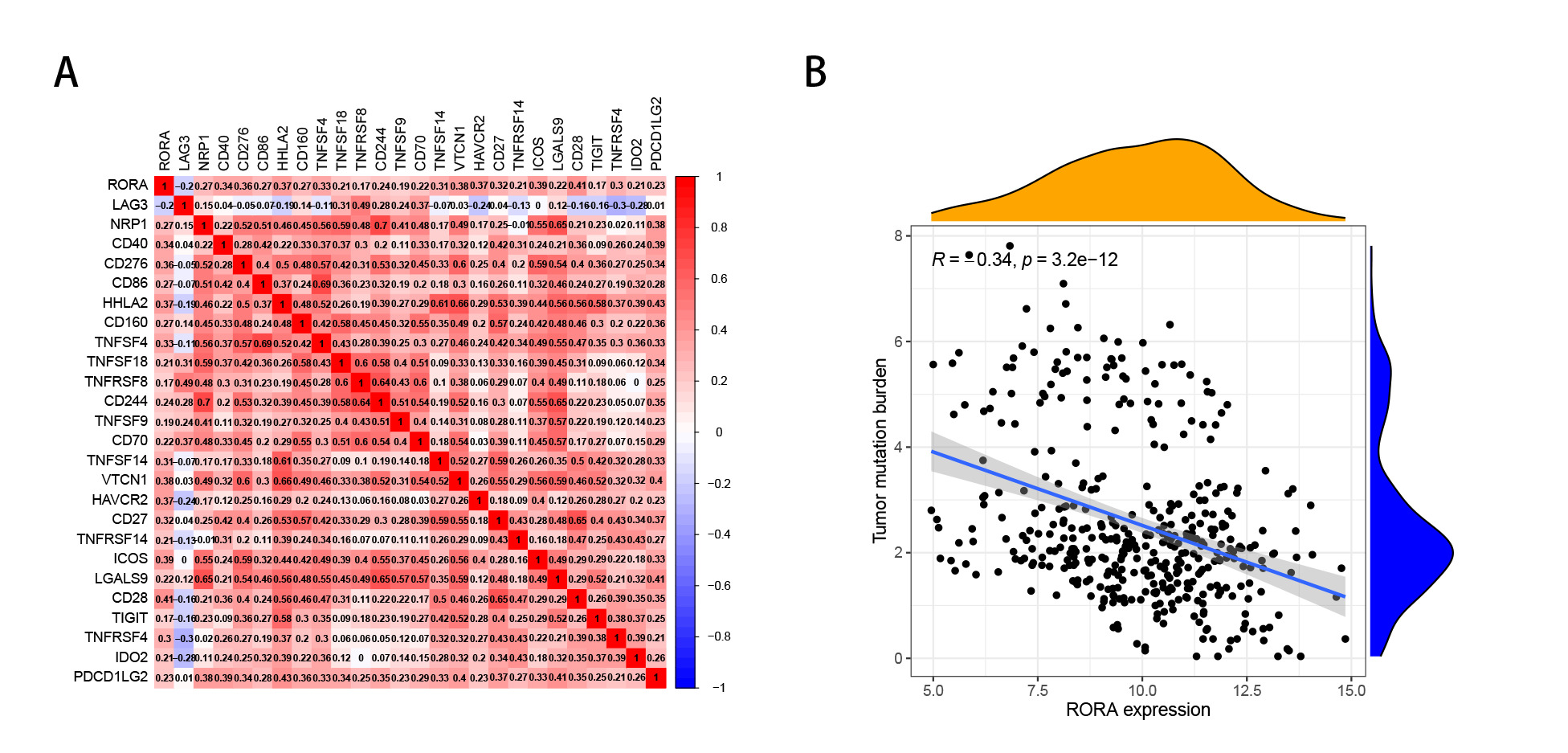

Supplement: S2 Fig — (TIF) [file pone.0327323.s002.tif]

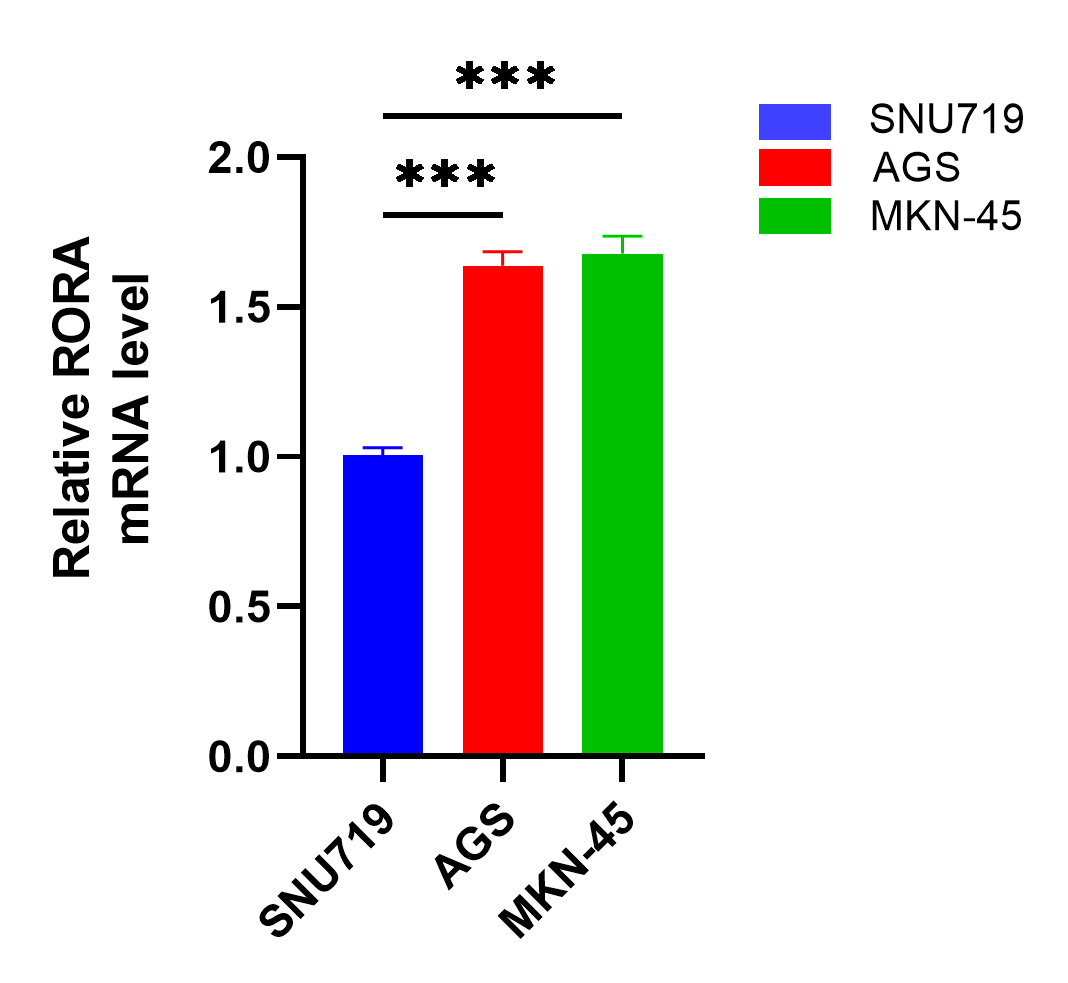

Supplement: S3 Fig — (TIF) [file pone.0327323.s003.tif]

AGS(RORA)



AGS(GAPDH)





MKN-45(RORA)





MKN-45(GAPDH)





SNU719(RORA)


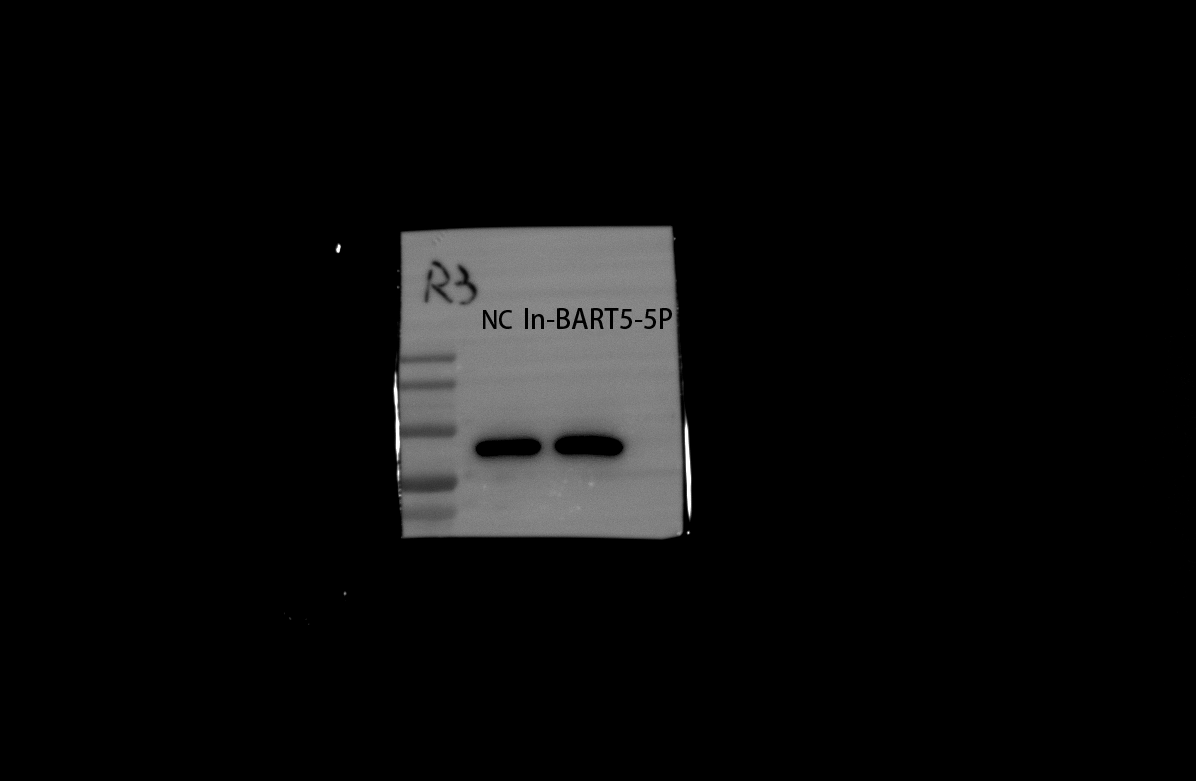


SNU719(GAPDH)


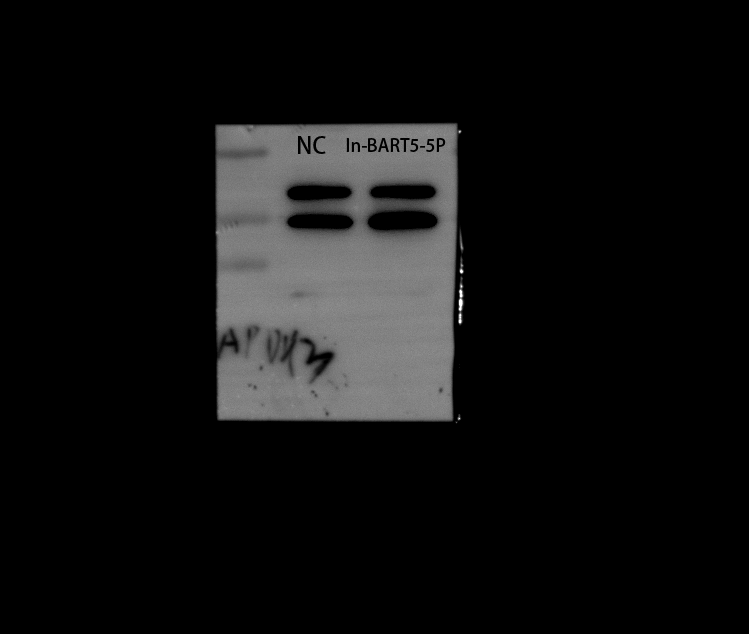

Supplement: S1 File — (DOCX) [file pone.0327323.s004.docx]
